# Retraction: miR-182-5p improves the viability, mitosis, migration and invasion ability of human gastric cancer cells by down-regulating RAB27A

**DOI:** 10.1042/BSR-2017-0136_RET

**Published:** 2021-09-22

**Keywords:** gastric cancer, miR-182-5p

This article is being retracted from *Bioscience Reports* at the request of the Editor-in-Chief and the Editorial Board following receipt of a notification from a reader, alerting the Editorial Board to a duplicated panel in Figure 4A. Post publication review by the Editorial Office also identified signs of image manipulation in the original version of Figure 4A as well as further duplications in the original version of 4C, which were replaced by the authors during the revision process.

The authors have been contacted in regards to the Retraction, and have not responded to the Journal's queries and the concerns raised. Given the extent of the issues raised, the Editorial Board stand by the decision to retract the article.

